# Supramolecular host-guest nanosystems for overcoming cancer drug resistance

**DOI:** 10.20517/cdr.2023.77

**Published:** 2023-11-22

**Authors:** Sha Wu, Miaomiao Yan, Minghao Liang, Wenzhi Yang, Jingyu Chen, Jiong Zhou

**Affiliations:** ^1^Department of Chemistry, College of Sciences, Northeastern University, Shenyang 110819, Liaoning, China.; ^2^Guangdong Provincial Key Laboratory of Functional Supramolecular Coordination Materials and Applications, Jinan University, Guangzhou 510632, Guangdong, China.

**Keywords:** Supramolecular nanosystems, host-guest interaction, cancer drug resistance

## Abstract

Cancer drug resistance has become one of the main challenges for the failure of chemotherapy, greatly limiting the selection and use of anticancer drugs and dashing the hopes of cancer patients. The emergence of supramolecular host-guest nanosystems has brought the field of supramolecular chemistry into the nanoworld, providing a potential solution to this challenge. Compared with conventional chemotherapeutic platforms, supramolecular host-guest nanosystems can reverse cancer drug resistance by increasing drug uptake, reducing drug efflux, activating drugs, and inhibiting DNA repair. Herein, we summarize the research progress of supramolecular host-guest nanosystems for overcoming cancer drug resistance and discuss the future research direction in this field. It is hoped that this review will provide more positive references for overcoming cancer drug resistance and promoting the development of supramolecular host-guest nanosystems.

## INTRODUCTION

With the number of cancer cases increasing each year, cancer has become the second leading cause of death worldwide^[1]^. Although chemotherapy remains the primary method of cancer treatment, its effectiveness is severely limited by cancer drug resistance^[2-5]^. The occurrence of cancer drug resistance is associated with multiple factors, including the overexpression of multidrug resistance gene (MDR1), anti-apoptotic protein (BCL-2), multidrug resistance-associated protein (MRP), and the enhanced activity of glutathione S-transferase (GST) and DNA repair enzyme^[6-8]^. These factors can lead to decreased drug uptake, increased drug efflux, DNA damage repair, abnormal drug metabolism, and dysfunctional apoptosis, resulting in cancer drug resistance^[9,10]^. Nanosystems have been widely used to overcome cancer drug resistance due to their ability to alter the way drugs enter cells, increase drug uptake, and improve drug stability^[11,12]^. Common nanosystems used to overcome cancer drug resistance include liposomes, polymeric nanoparticles, and metal nanoparticles. However, there are still some problems in the application of these nanosystems. For example, drugs loaded in liposomes tend to leak in the circulatory system before reaching the tumor; polymeric nanoparticles have a high burst release effect; and metal nanoparticles have poor biocompatibility. These problems have led to the limited role of these nanosystems in overcoming cancer drug resistance^[13,14]^. Therefore, it is urgent to develop a class of novel nanosystems to reverse cancer drug resistance.

Supramolecular chemistry is “chemistry beyond the molecule”^[15]^. Supramolecules generally refer to organized aggregates formed by non-covalent interactions of two or more molecules, including electrostatic interaction, hydrogen bond, van der Waals force, and π-π interaction^[16-21]^. By introducing supramolecules into the nanosystem, it is possible to construct a more promising new drug delivery system, supramolecular host-guest nanosystem, which provides a potential solution for cancer drug resistance^[22-28]^. Compared with traditional nanomaterials constructed by covalent interactions, supramolecular host-guest nanomaterials constructed by non-covalent interactions have excellent dynamic reversibility and responsiveness to various stimuli (such as weak acidity, specific enzymes, and different redox environments)^[29-33]^. Based on these advantages, supramolecular host-guest nanosystems can increase drug uptake, accurately release drugs, inhibit drug efflux, and protect the activity of drugs, which provide great possibilities for eliminating cancer drug resistance and promoting the progress of cancer treatment^[34-37]^.

**Scheme 1:**
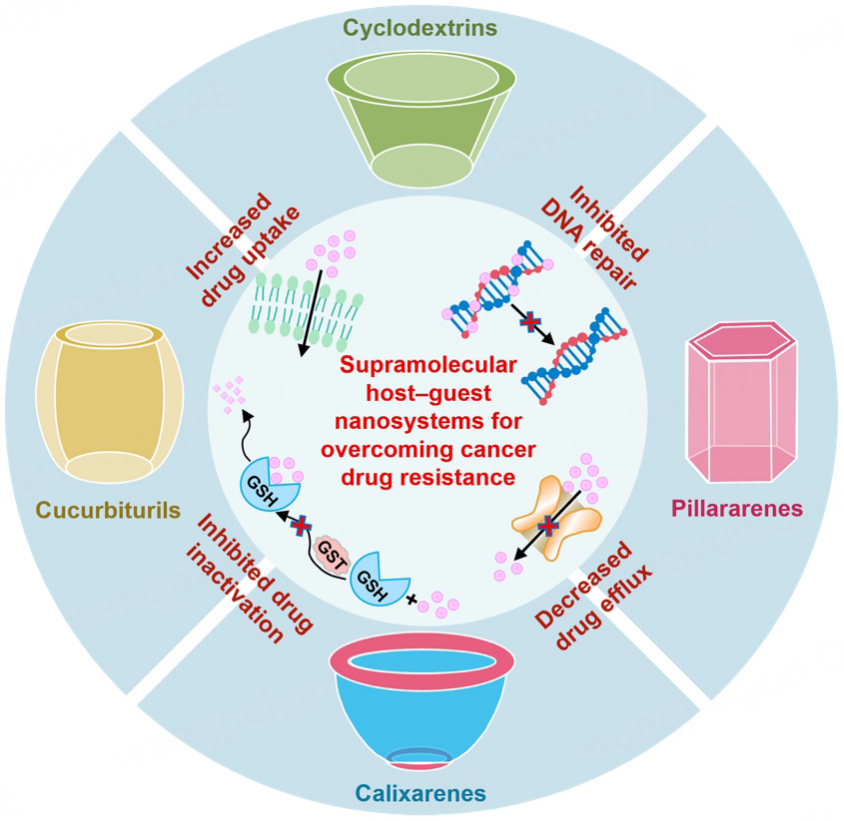
Supramolecular host-guest nanosystems for overcoming cancer drug resistance. GSH: Glutathione; GST: glutathione S-transferase.

In this review, we summarize the research progress of supramolecular host-guest nanosystems for overcoming cancer drug resistance over the past few years, including cyclodextrins, calixarenes, cucurbiturils, and pillararenes [Scheme 1]. Moreover, the challenges and prospects of supramolecular host-guest nanosystems for overcoming cancer drug resistance are discussed extensively. This review aims to provide valuable insights and contribute to the development of more effective ways to reverse cancer drug resistance.

## CYCLODEXTRINS-BASED HOST-GUEST NANOSYSTEMS FOR OVERCOMING CANCER DRUG RESISTANCE

**Figure 1:**
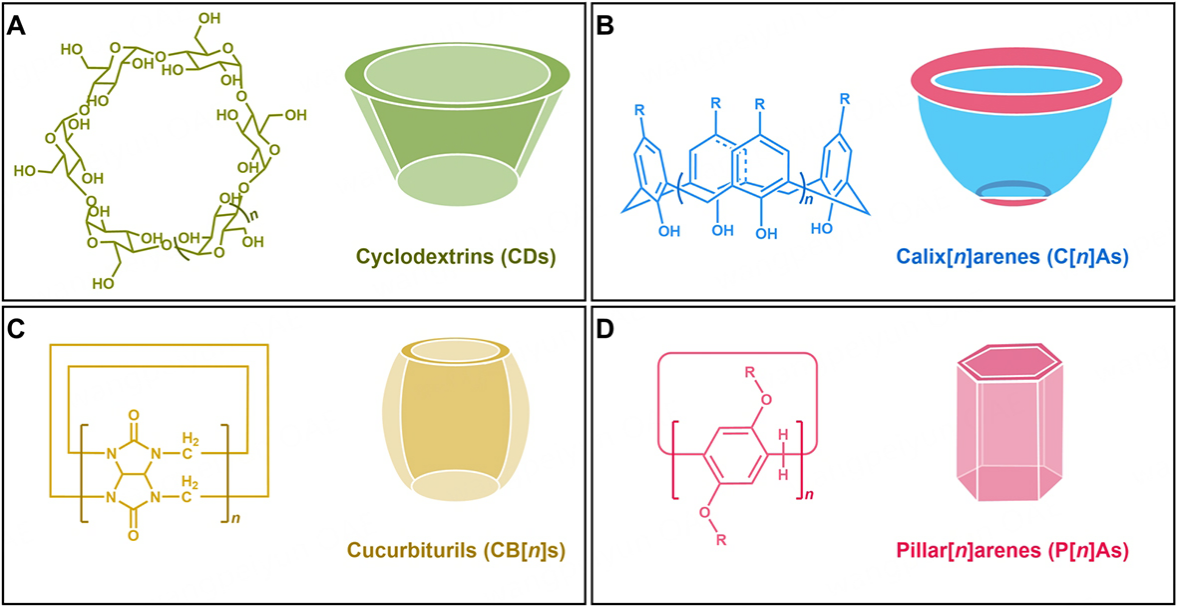
Schematic illustration of structures of (A) CDs; (B) calixarenes (C[*n*]As); (C) cucurbiturils (CB[*n*]s); and (D) pillararenes (P[*n*]As).

Cyclodextrins (CDs), a class of natural oligosaccharides obtained from the degradation of starch, are linked by glucopyranose units through α-1,4-glycosidic bonds [Figure 1]^[38,39]^. The most common CDs contain six, seven, and eight glucopyranose units, respectively, known as α, β, and γ-CDs^[40,41]^. CDs have hydrophobic cavities, which can encapsulate hydrophobic drug molecules to form host-guest complexes^[42-45]^. In addition, these complexes can self-assemble into nanoparticles, greatly improving the efficiency of the drug (such as good water solubility, high stability, and low physiological toxicity)^[46-48]^. Therefore, CDs-based host-guest nanosystems have the potential to reverse cancer drug resistance by increasing drug uptake and decreasing drug efflux^[49-51]^. For example, Yang *et al.* constructed three nanomedicines based on β-CDs that enhanced the drug uptake and the toxicity of drug-resistant cells^[52]^. Das *et al.* prepared a dual-responsive nanocarrier by embedding carbon nanotubes into β-CDs-based polymers, enabling the combination of cocktail chemotherapy with photothermal therapy, which was conducive to multidrug resistance reversal^[53]^.

**Figure 2:**
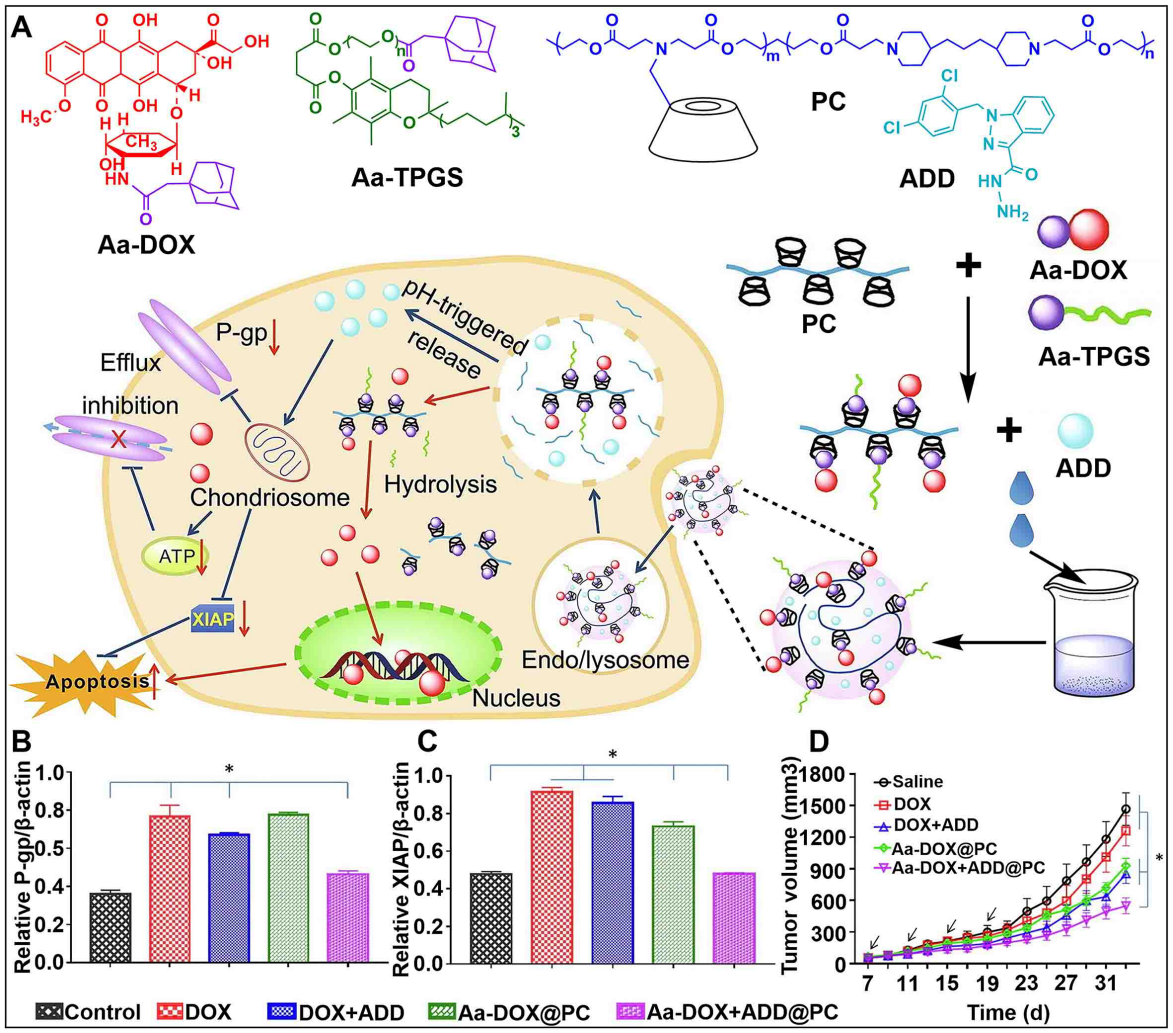
(A) Schematic illustration of dual-drug co-loaded nanoparticle (Aa-DOX + ADD@PC) for overcoming cancer drug resistance; The expression levels of (B) P-gp and (C) XIAP in MCF-7/ADR cells with different treatments; (D) Tumor growth inhibition curves of tumor-bearing mice after various formulations. (^*^P < 0.05) This figure is quoted with permission from Wang *et al.*^[59]^. ADD: Mitochondrial inhibitor; DOX: doxorubicin; XIAP: X-linked inhibitor of apoptosis protein.

P-glycoprotein (P-gp) is an energy-dependent efflux pump located on the cell membrane^[54,55]^. P-gp depends on the energy produced by ATP hydrolysis within the mitochondria to keep intracellular drug concentrations low by transporting drug molecules outside the cell, resulting in drug resistance^[56-58]^. Therefore, drug resistance can be effectively reversed by inducing mitochondrial dysfunction. Wang *et al.* constructed a nanosystem (Aa-DOX + ADD@PC) based on a pH-sensitive graft copolymer (PBAE-g-β-CD) to achieve co-loading of the anticancer drug doxorubicin (DOX) and mitochondrial inhibitor (ADD) [Figure 2A]^[59]^. When Aa-DOX + ADD@PC was endocytosed by tumor cells, DOX and ADD were released in the acidic environment for combined chemotherapy. Western blot assay was used to study the expression levels of P-gp and X-linked inhibitor of apoptosis protein (XIAP), and it was found that Aa-DOX + ADD@PC showed the best inhibitory effect on P-gp and XIAP [Figures 2B and C]. Moreover, the therapeutic effect of Aa-DOX + ADD@PC was better than that of free DOX, significantly inhibiting the growth of drug-resistant tumors [Figure 2D]. In this work, the effective loading of mitochondrial inhibitors by CDs was used to successfully reverse drug resistance by decreasing drug efflux, providing a new therapeutic platform for overcoming multidrug resistance (MDR).

**Figure 3:**
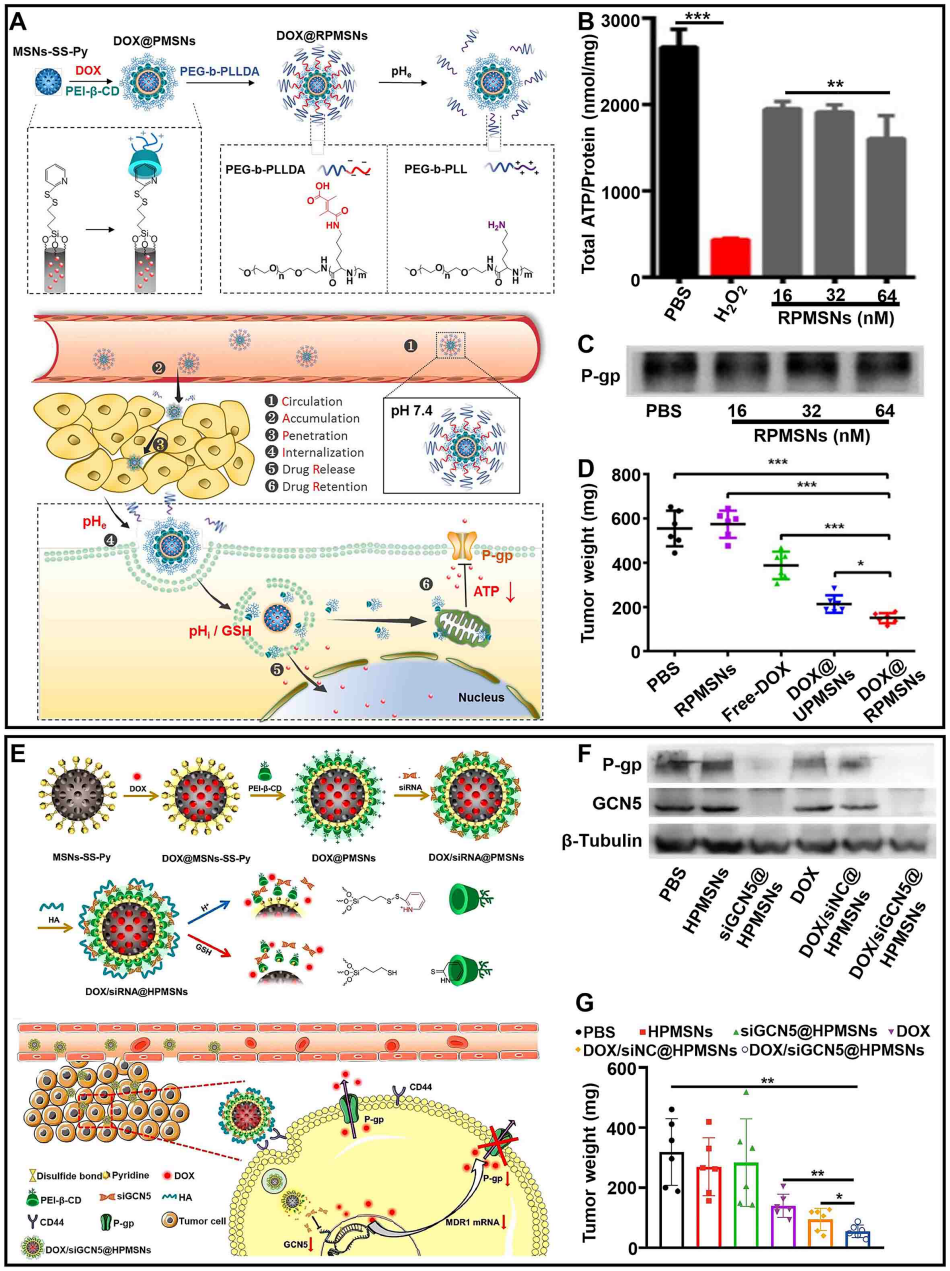
(A) Schematic illustration of the construction of sequentially responsive nanosystem (DOX@RPMSNs) and dual-responsive drug release; (B) Total ATP concentrations of MCF7/ADR cells treated with H_2_O_2_ and different doses of RPMSNs; (C) The expression levels of P-gp in tumor cells with different treatments; (D) Changes of tumor volume in tumor-bearing mice with different treatments (^*^P < 0.05, ^**^P < 0.01, ^***^P < 0.001). This figure is quoted with permission from Liu *et al.*^[62]^; (E) Schematic illustration of the construction of co-delivery nanosystem (HPMSNs) and dual-responsive drug release; (F) The expression levels of P-gp and GCN5 in tumor cells with different treatments; (G) Changes of tumor weight in tumor-bearing mice with different treatments (^*^P < 0.05, ^**^P < 0.01). This figure is quoted with permission from Yuan *et al.*^[68]^. DOX: Doxorubicin; PEG-b-PLLDA: poly (ethylene glycol) amine derivative shell.

Furthermore, compared with free drugs, tumor cells can effectively take up nanomedicine, which is conducive to the reversal of drug resistance caused by low intracellular drug concentration^[60,61]^. Liu *et al.* developed pH/redox dual-responsive DOX delivery nanosystems (DOX@RPMSNs) based on cationic β-cyclodextrin-PEI (PEI-β-CD) to overcome drug resistance of tumor cells [Figure 3A]^[62]^. The poly (ethylene glycol) amine derivative shell (PEG-b-PLLDA) of DOX@RPMSNs could protect DOX@RPMSNs from safely reaching the vicinity of tumor cells, increasing the absorption of drugs by tumor cells. PEI-β-CD and DOX were sequentially released in response to the action of acid and glutathione (GSH) in tumor cells. DOX was used to kill tumor cells, and PEI-β-CD acted as an inhibitor to downregulate the expression of drug resistance-related P-gp by reducing ATP [Figure 3B]. Compared with other formulations, DOX@RPMSNs significantly inhibited tumor growth [Figures 3C and D]. These results indicated that DOX@RPMSNs successfully improved drug resistance reversal.

Histone-acetyltransferase (GCN5) is a silencing protein closely related to drug-resistance genes. Drug resistance caused by efflux can be reversed by down-regulating the expression of GCN5^[63,64]^. RNA interference (RNAi) is a therapeutic technique that specifically targets mRNA and regulates the expression of silencing proteins^[65-67]^. Yuan *et al.* exploited a nanosystem (DOX/siRNA@HPMSNs) to combine RNAi and DOX, which could knockout drug-resistance genes (Figure 3E)^[68]^. The hyaluronan (HA) shell of DOX/siRNA@HPMSNs could prolong the circulation time of DOX/siRNA@HPMSNs *in vivo* and target tumor cells, which promoted the accumulation of antitumor drugs. In the microenvironment of tumor cells, the effective release of siRNA could downregulate the expression of GCN5 to reduce the efflux of DOX caused by P-gp [Figure 3F]. Additionally, the inhibition rate of DOX/siRNA@HPMSNs on the growth of drug-resistance tumors was higher than DOX by evaluating the chemotherapeutic effects of different drug delivery systems [Figure 3G]. The two pH/redox dual-responsive nanosystems reduced drug efflux caused by the overexpression of P-gp in different ways, providing more possibilities for reversing MDR.

**Figure 4:**
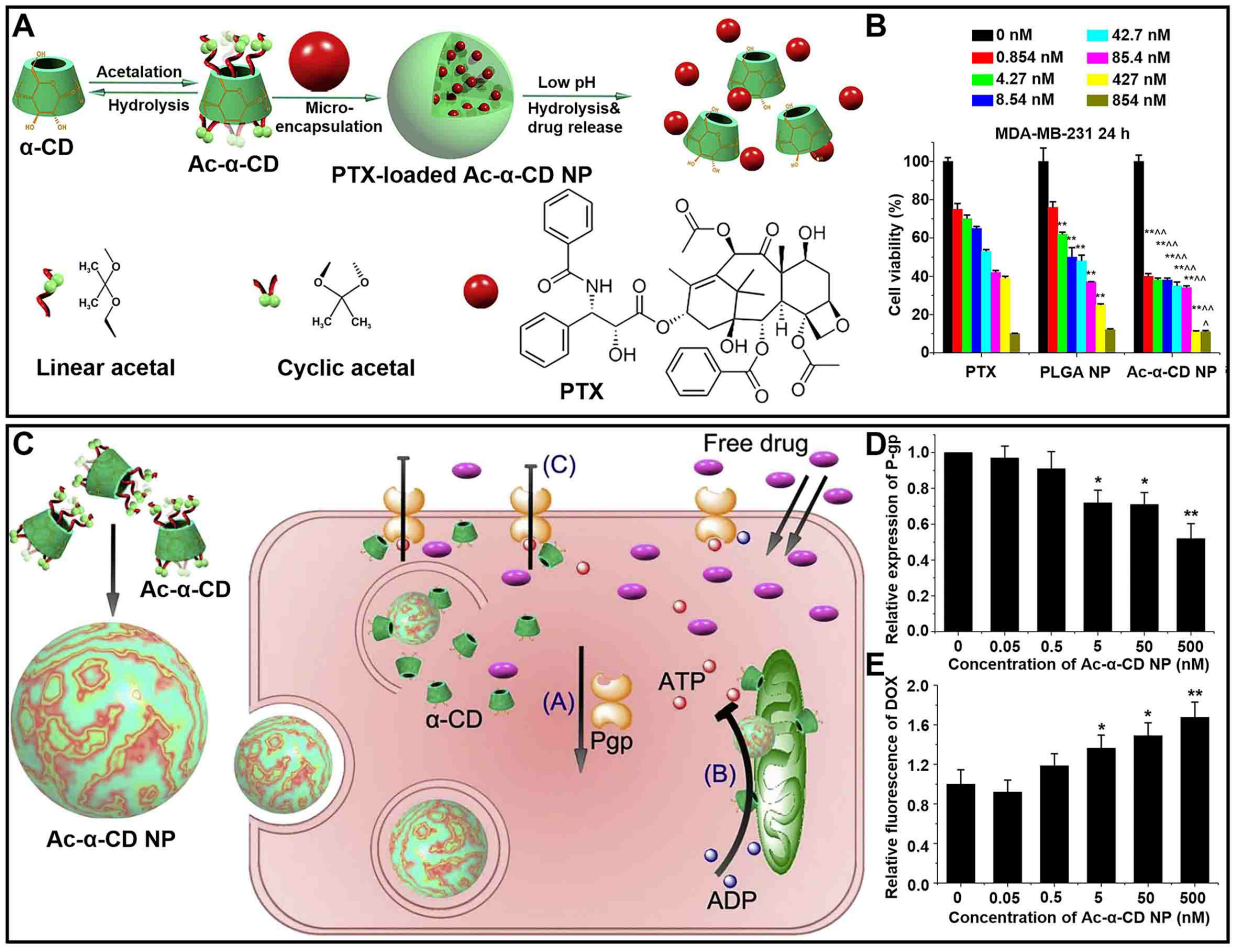
(A) Schematic illustration of the formation of pH-sensitive nanosystems (Ac-α-CD NP); (B) The cell viability of MDA-MB-231 cells treated with different doses of PTX, PLGA NP, and Ac-α-CD NP (^**^P < 0.01). This figure is quoted with permission from He *et al.*^[74]^; (C) Schematic illustration of the antitumor progress of Ac-α-CD NP in MCF-7/ADR cells; (D) The P-gp expression level; and (E) the accumulation of DOX with different doses of Ac-α-CD NP in MCF-7/ADR cells (^*^P < 0.05, ^**^P < 0.01). This figure is quoted with permission from Shi *et al.*^[75]^. CD: Cyclodextrin; DOX: doxorubicin; NP: nanoparticle; PTX: paclitaxel.

The acidic tumor microenvironment commonly found in solid tumors can reduce the endocytosis of free drugs and dissociate drug molecules^[69,70]^. Therefore, pH-responsive supramolecular host-guest nanosystems have been widely developed to enhance cell internalization and protect drugs from dissociation^[71-73]^. He *et al.* prepared a pH-responsive nanoparticle (Ac-α-CD NP) based on acetylated α-CD (Ac-α-CD), which could stably encapsulate paclitaxel (PTX) [Figure 4A]^[74]^. Ac-α-CD NP had a stronger inhibitory effect on the viability of breast cancer drug-resistant cells (MDA-MB-231) compared with free PTX and PLGA NPs [Figure 4B]. Moreover, Ac-α-CD NP exhibited good drug activity at a low concentration (0.854 nM), indicating its potential to kill drug-resistant cells.

Additionally, further studies showed that Ac-α-CD NP could also enhance the uptake and sensitivity of drug-resistant cells to DOX [Figure 4C]^[75]^. α-CD was released by pH-induced hydrolysis of Ac-α-CD NP to inhibit the expression of P-gp and decrease the activity of ATPase, eliminating drug resistance caused by drug efflux [Figure 4D]. The changes in drug concentrations indicated that the downregulation of P-gp expression directly increased the accumulation of DOX in drug-resistant cells, thus achieving the purpose of inhibiting cancer drug resistance [Figure 4E]. Such pH-responsive supramolecular nanoparticles could not only inhibit the viability of drug-resistant cells at low concentrations but also increase the uptake and sensitization of drug-resistant cells to DOX, successfully reversing drug resistance from multiple angles.

Various star-shaped polymers can be obtained by modifying β-CD with different polymer chains^[76,77]^. These polymers can further self-assemble into stable supramolecular host-guest nanoparticles after loading anticancer drugs^[78-80]^. Compared with free drugs, nanoparticles are more easily internalized by tumor cells, reducing the efflux of drugs^[81-83]^. These factors work together to eliminate cancer drug resistance^[84,85]^. Chen *et al.* constructed a cationic β-CD-based nanocarrier that co-delivered PTX and Nur77 gene (an orphan nuclear receptor) to eliminate cancer drug resistance^[86]^. In addition, they reported a nanoparticle based on a PEGylated star-shaped copolymer successfully reversed MDR1-induced drug resistance^[87]^.

**Figure 5:**
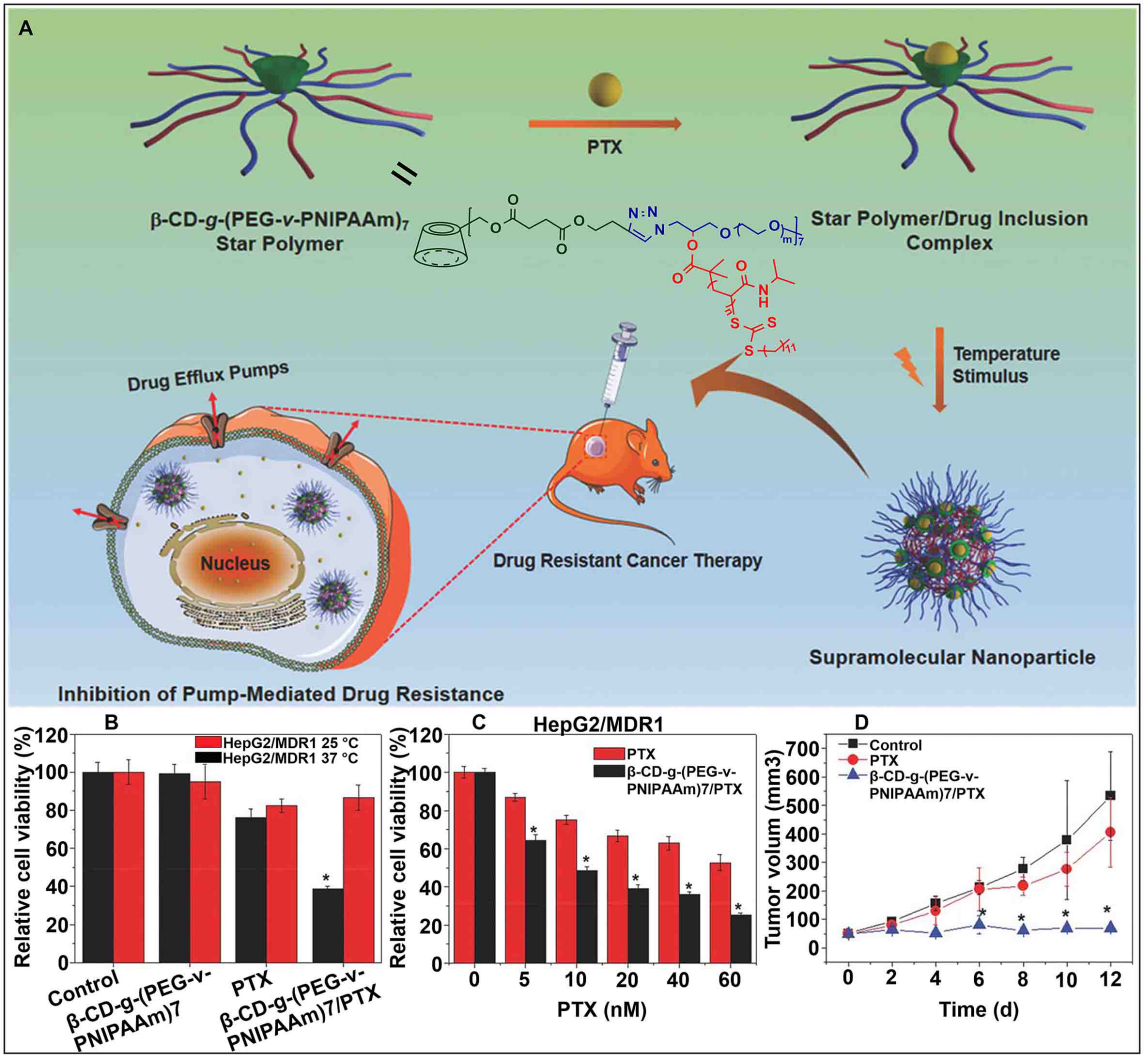
(A) Schematic illustration of supramolecular nanoparticle for inhibiting pump-mediated drug resistance; (B) Cell viability of HepG2/MDR1 cells treated with β-CD-*g*-(PEG-*v*-PNIPAAm)_7_, PTX, and β-CD-*g*-(PEG-*v*-PNIPAAm)_7_/PTX at different temperatures; (C) Changes in cell viability of HepG2/MDR1 cells treated with different doses of PTX and β-CD-*g*-(PEG-*v*-PNIPAAm)_7_/PTX; (D) Tumor growth inhibition curves of tumor-bearing mice after various treatments (^*^P < 0.05). This figure is quoted with permission from Fan *et al.*^[88]^. CD: Cyclodextrin; MDR1: multidrug resistance gene; PEG: poly (ethylene glycol); PTX: paclitaxel.

Subsequently, they designed a new type of thermosensitive star-shaped polymer β-CD-*g*-(PEG-*v*-PNIPAAm)_7_ with “V”-shaped arms, which encapsulated PTX through the cavity of β-CD [Figure 5A]^[88]^. The drug-loaded polymer further self-assembled into a stable supramolecular host-guest nanomedicine at 37 °C, greatly enhancing the retention of drugs in cells. Compared with other drugs, this supramolecular host-guest nanomedicine was more sensitive to the change of temperature, causing a sharp decline in cell viability at 37 °C [Figure 5B]. When the drug-resistance tumor was transplanted into mice and treated with PTX and nanomedicine, respectively, β-CD-*g*-(PEG-*v*-PNIPAAm)_7_/PTX was more prominent in reducing cell viability [Figure 5C]. Additionally, there was no obvious change in tumor volume after treatment with β-CD-*g*-(PEG-*v*-PNIPAAm)_7_/PTX [Figure 5D]. These results indicated that β-CD-based temperature-responsive nanomedicine had a good therapeutic efficacy against drug-resistant tumors.

**Figure 6:**
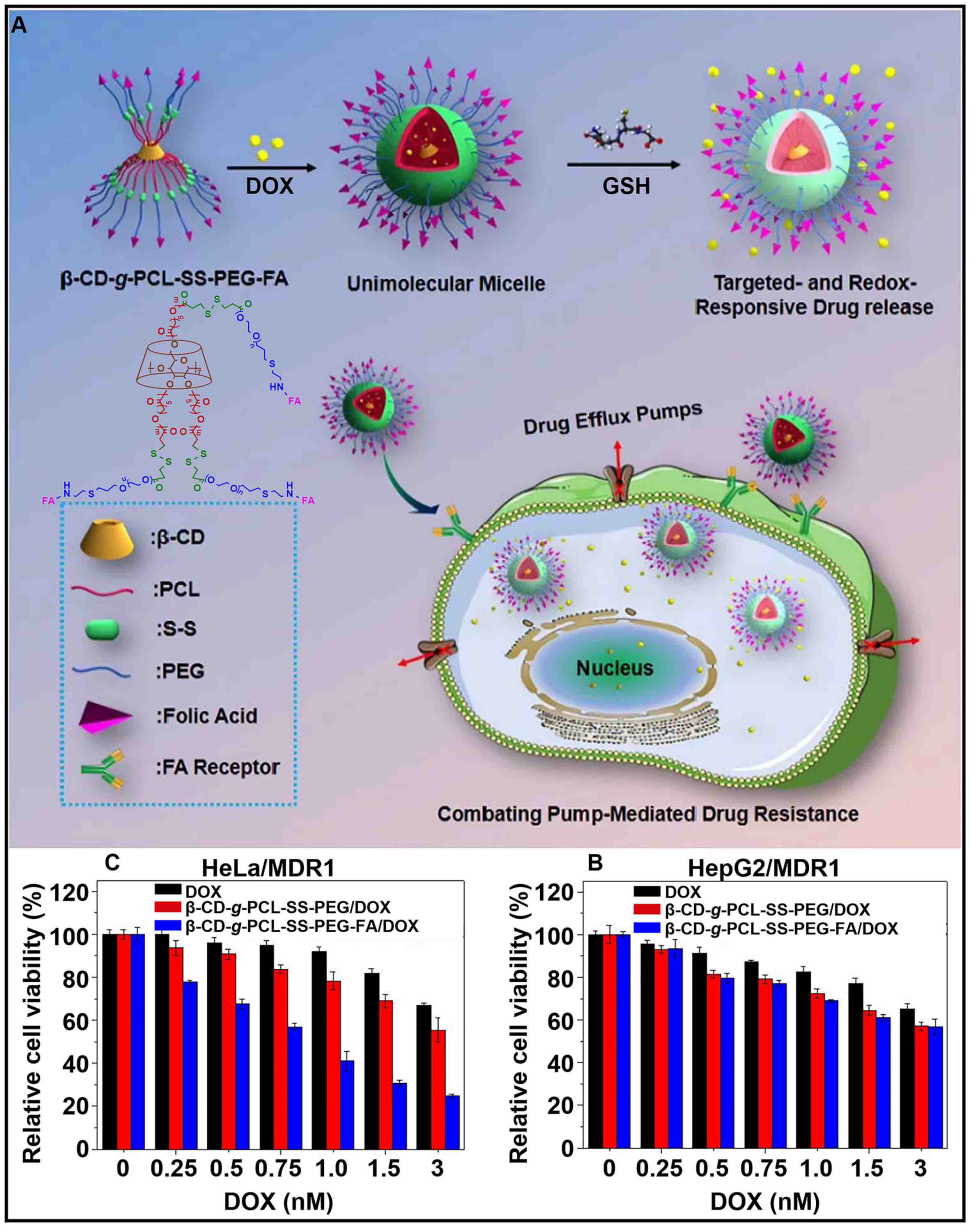
(A) Schematic illustration of a unimolecular micelle for inhibiting pump-mediated drug resistance; Cell viability of (B) HepG2/MDR1 cells and (C) HeLa/MDR1 cells treated with different doses of DOX, β-CD-*g*-PLC-SS-PEG/DOX, and β-CD-*g*-PLC-SS-PEG-FA/DOX. This figure is quoted with permission from Li *et al.*^[89]^. CD: Cyclodextrin; DOX: doxorubicin; FA: folic acid; GSH: glutathione; MDR1: multidrug resistance gene; PEG: poly (ethylene glycol).

Moreover, Li *et al.* constructed a unimolecular micelle based on a star-shaped polymer (β-CD-*g*-PCL-SS-PEG-FA) that stably encapsulated DOX [Figure 6A]^[89]^. The folic acid (FA) in the unimolecular micelle could target and penetrate tumor cells to increase the accumulation of DOX, inhibiting the cancer drug resistance caused by decreased drug uptake. The drug loaded in the unimolecular micelle could be released in response to GSH. MTT assay analysis indicated that β-CD-*g*-PCL-SS-PEG-FA had a better inhibitory effect on cell viability compared with free DOX [Figure 6B and C]. In addition, the overexpression of folate receptors on cervical cancer drug-resistant cells (HeLa/MDR1) accelerated the uptake of β-CD-*g*-PCL-SS-PEG-FA, enhancing the therapeutic effect of DOX on drug-resistant cells. Such β-CD-based stimuli-responsive supramolecular host-guest nanoparticles showed exciting results in overcoming cancer drug resistance due to the precise targeting, effective uptake, and controlled release of drugs.

## CALIXARENES-BASED HOST-GUEST NANOSYSTEMS FOR OVERCOMING CANCER DRUG RESISTANCE

Calixarenes are a class of cyclic oligomers formed by methylene-bridging ortho-phenolic hydroxyl groups [Figure 1B]^[90-92]^. Due to their molecular shapes similar to the Sangreal, they are named calix[*n*]arenes (C[*n*]As) by Gutsche^[93]^. By introducing hydrophilic and hydrophobic groups at the upper and lower rims of C[*n*]As, respectively, amphiphilic C[*n*]As can be designed, which are easy to self-assemble into vesicles, nanoparticles, or other aggregates^[94-99]^. Host-guest nanosystems based on C[*n*]As have low toxicity and good biocompatibility, becoming a new research hotspot in the field of cancer drug resistance^[100-102]^.

**Figure 7:**
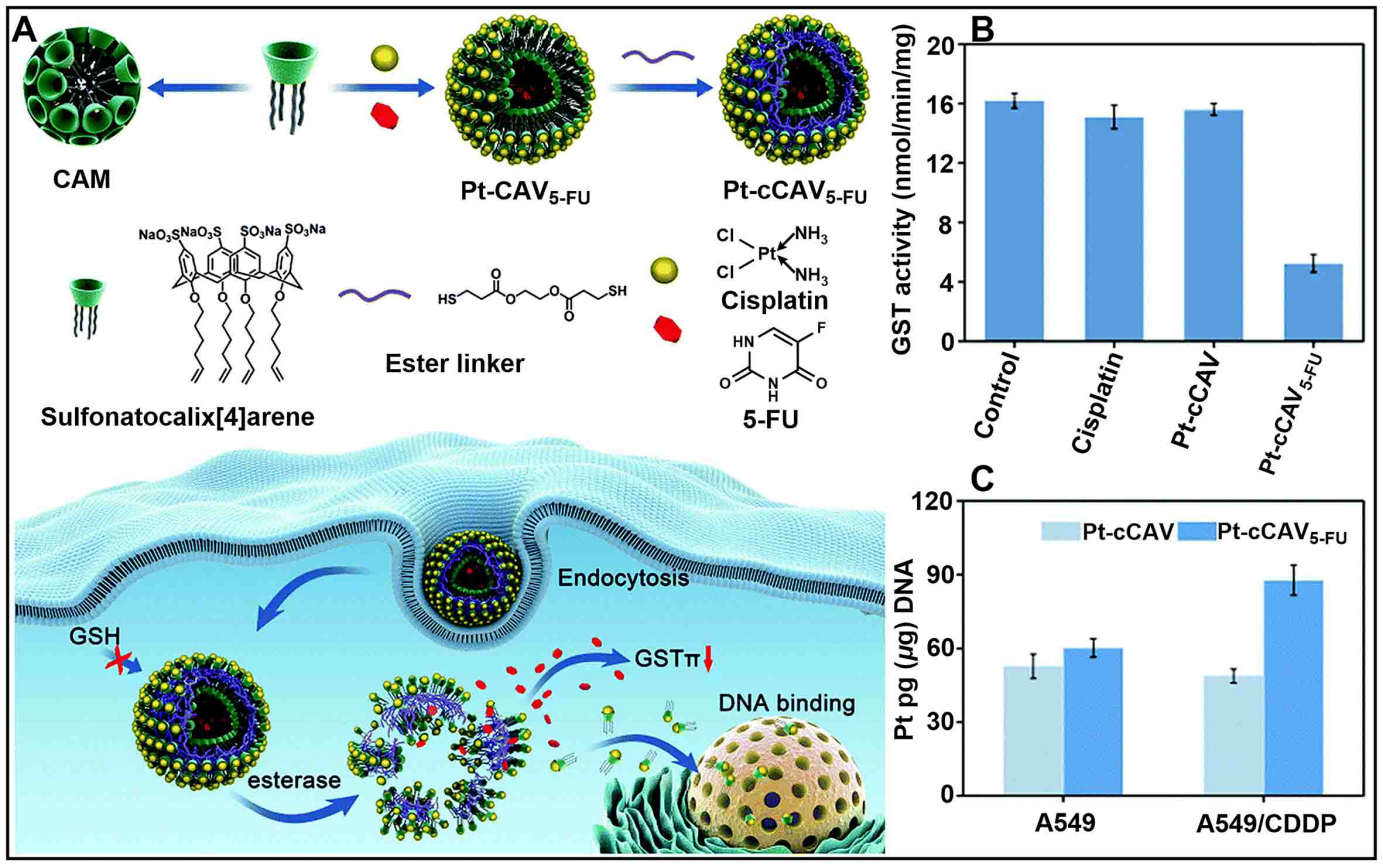
(A) Schematic illustration of Pt-cCAV_5-FU_ for overcoming cisplatin resistance in A549/CDDP cells; (B) The GST activity of A549/CDDP cells with different treatments; (C) Platinum content in the genomic DNA of A549 and A549/CDDP cells after incubation with Pt-cCAV and Pt-cCAV_5-FU_ for 12 h. This figure is quoted with permission from Dai *et al.*^[109]^. GSH: Glutathione; GST: glutathione S-transferase.

The close coordination between GSH and GST can initiate detoxification mechanisms within tumor cells, leading to the formation of drug resistance^[103-105]^. For example, GST can catalyze the binding of GSH to electrophilic antitumor drugs, accelerating the degradation of drugs^[106-108]^. Therefore, drug resistance can be reversed by regulating the GST. Recently, Dai *et al.* designed a nanomedicine (Pt-cCAV_5-FU_) based on sulfonatocalix[4]arene for overcoming GST-induced cancer drug resistance [Figure 7A]^[109]^. The GST regulator (5-FU) was encased into the hydrophilic core of Pt-cCAV_5-FU_ self-assembled from a host-guest complex of sulfonatocalix[4]arene with cisplatin. Pt-cCAV_5-FU_ actively released cisplatin and 5-FU during the hydrolysis process caused by esterase. 5-FU could downregulate GST activity, prompting cisplatin to damage DNA rather than binding to GSH [Figure 7B]. In addition, the endocytosis of cisplatin resistance cells A549/CDDP against Pt-cCAV_5-FU_ was stronger than that of A549, which increased the accumulation of cisplatin in cancer cells [Figure 7C]. All of these factors ultimately made Pt-cCAV_5-FU_ more toxic to drug-resistance cells. This work developed a novel nanomedicine, laying the foundation for C[*n*]As-based host-guest nanosystems to reverse cisplatin resistance.

**Figure 8:**
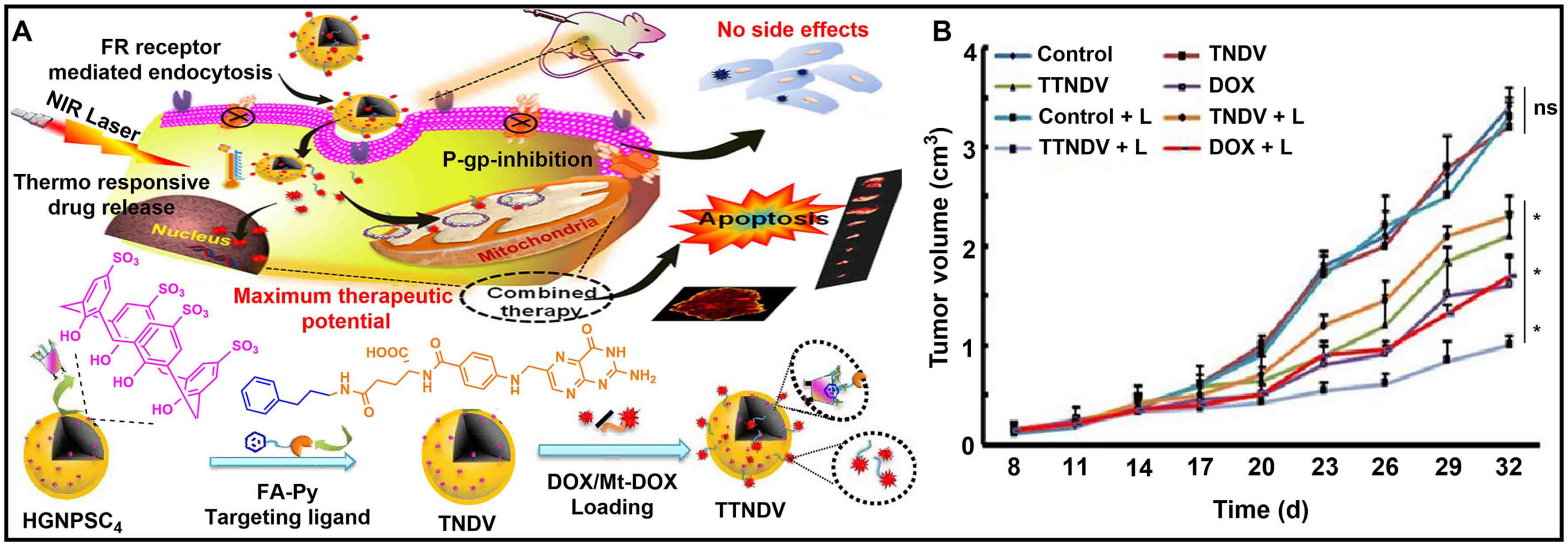
(A) Schematic illustration of TTNDV to overcome cancer drug resistance by killing n-DNA and Mt-DNA; (B) Tumor growth inhibition curves of tumor-bearing mice after different formulations (^*^P < 0.05). This figure is quoted with permission from Nair *et al.*^[114]^. DOX: Doxorubicin; FA: folic acid.

Furthermore, although free DOX can kill the nuclear DNA (n-DNA) of tumor cells, the undamaged mitochondrial DNA (Mt-DNA) can trigger drug resistance^[110-112]^. Therefore, the design of a drug to destroy synchronously n-DNA and Mt-DNA can promote the reversal of drug resistance^[113]^. Nair *et al.* constructed a gold nanotherapy platform (TTNDV) based on sulfonatocalix[4]arene [Figure 8A]^[114]^. The nanoplatform could encapsulate DOX and mitochondrion-targeted analogue (Mt-DOX) in an optimal ratio of 1:100 to reverse cancer drug resistance caused by mitochondrial escape. *In vitro* and *in vivo* experiments showed that TTNDV had less toxic side effects than free DOX. In addition, TTNDV had a stimulating response to temperature. Under near-infrared irradiation, drugs embedded in TTNDV were released simultaneously to kill n-DNA and Mt-DNA, successfully overcoming DOX resistance and improving the chemotherapeutic effect of DOX [Figure 8B]. This work solved the problem of DOX resistance by killing Mt-DNA to induce apoptosis, providing a feasible strategy for reversing cancer drug resistance.

## CUCURBITURILS-BASED HOST-GUEST NANOSYSTEMS FOR OVERCOMING CANCER DRUG RESISTANCE

Cucurbiturils (CB[*n*]s) are a kind of macrocyclic hosts constructed by condensation of glycoluril with formaldehyde under acid conditions, which are the fourth macrocyclic hosts after crown ethers, cyclodextrins, and calixarenes [Figure 1C]^[115-117]^. According to the different number of glycoluril units, different types of CB[*n*]s can be obtained, and common cucurbiturils include CB[6], CB[7], and CB[8]^[118,119]^. Due to their unique structures of hydrophobic cavity and hydrophilic port, CB[*n*]s are easy to form host-guest complexes with drug molecules and are promising materials for reducing side effects and enhancing the stability of antitumor drugs^[120-124]^. In addition, CB[*n*]s-based supramolecular nanosystems can be used to effectively reverse cancer drug resistance^[125-128]^.

**Figure 9:**
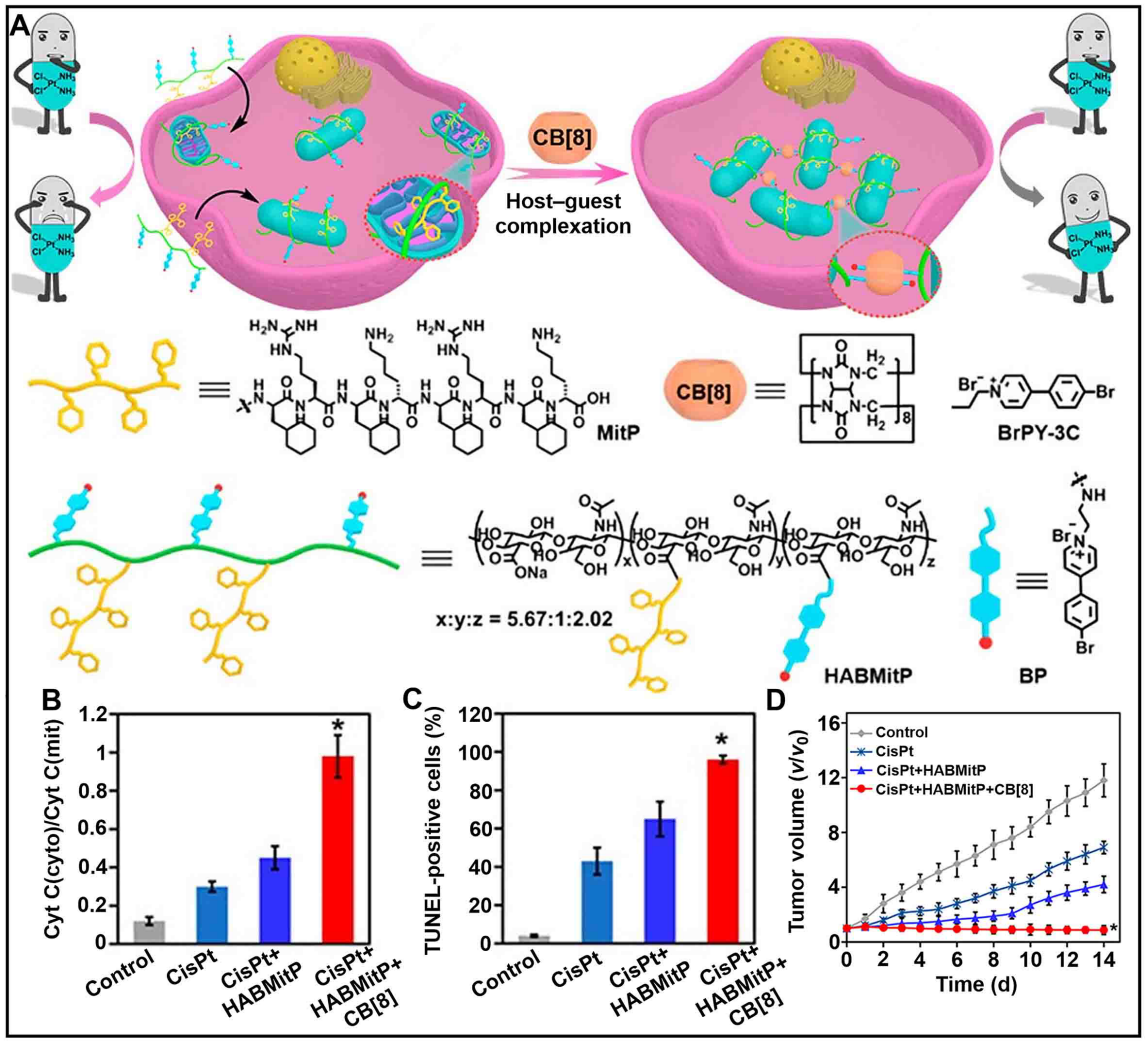
(A) Schematic illustration of mitochondrial aggregation progress after treatment with multivalent supramolecular polymer (HABMitP) and CB[8]; (B) The ratio of cytosol cytochrome C [Cyt C(cyto)] to mitochondrial cytochrome C [Cyt C(mit)] and (C) The apoptosis percentage of TUNEL-positive cells with different treatments; (D) Changes of tumor volume in tumor-bearing mice with different formulations (^*^P < 0.05). This figure is quoted with permission from Dai *et al.*^[136]^.

Cancer drug resistance is closely related to the inhibition of tumor apoptosis^[129,130]^. Mitochondria serves as the center for regulating tumor cell apoptosis^[131,132]^. Therefore, the destruction of mitochondria is also an effective way to overcome cancer drug resistance^[133-135]^. Recently, Dai *et al.* synthesized a multivalent supramolecular polymer (HABMitP) by modifying HA with mitochondrial targeting peptide and 4-bromophenylpyridium [Figure 9A]^[136]^. The combination of HABMitP, cisplatin, and CB[8] could promote mitochondrial aggregation, which led to the deterioration of mitochondria to release apoptosis-inducing factor (cytochrome C), thereby activating the apoptosis of tumor cells [Figure 9B and C]. Moreover, cisplatin-resistant tumors did not grow treated with CisPt + HABMitP + CB[8] for 14 days, indicating that assembly-induced mitochondrial aggregation significantly improved the antitumor efficacy of cisplatin [Figure 9D]. This study showed that the regulation of mitochondrial behavior was beneficial to the reversal of drug resistance, which provided a broad prospect for overcoming tumor drug resistance.

**Figure 10:**
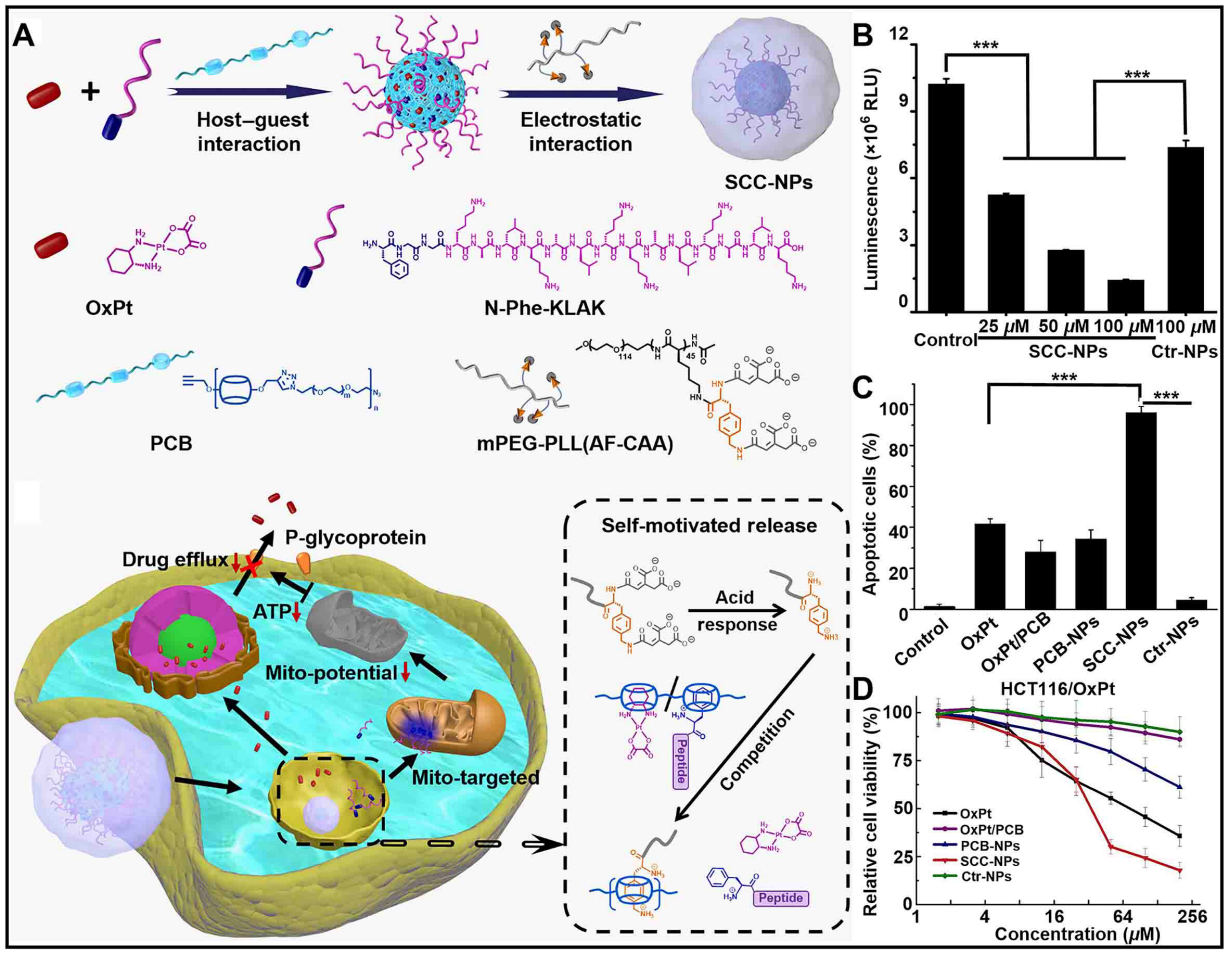
(A) Schematic illustration of the preparation and mechanism of self-motivated nanoparticles (SCC-NPs) in overcoming drug resistance; (B) ATP levels in HCT116/OxPt cells treated with Ctr NPs and SCC NPs at different doses; (C) The number of apoptotic cells treated with different formulations; (D) Cell viability of HCT116/OxPt cells after incubating with different treatments (^***^P < 0.001). This figure is quoted with permission from Wang *et al.*^[139]^. PEG: Poly (ethylene glycol).

The inhibition of P-gp expression by reducing ATP concentration can reduce drug resistance in tumor cells^[137,138]^. Wang *et al.* reported nanoparticles (SCC-NPs) based on CB[7], which encapsulated the anticancer drug oxaliplatin (OxPt) and mitochondria-targeting peptide (N-Phe-KLAK) by the excellent host-guest properties of CB[7] [Figure 10A]^[139]^. Due to the special acid responsiveness and competitiveness of the polymeric shell, SCC-NPs were used for self-motivated supramolecular combination chemotherapy. In acidic tumor environments, the amidomethyl phenylamine moieties on the polymeric shell were restored to form host-guest complexes with CB[7], competing to replace and release OxPt and N-Phe-KLAK. The released N-Phe-KLAK could effectively inhibit the production of ATP, resulting in the damage of energy-dependent drug efflux pump [Figure 10B]. Additionally, the accumulation of OxPt in cells directly led to an increase in the number of apoptotic cancer cells, which successfully inhibited the viability of drug-resistance cells [Figure 10C and D). Self-motivated supramolecular combination chemotherapy provided a new strategy for addressing the issue of cancer drug resistance.

**Figure 11:**
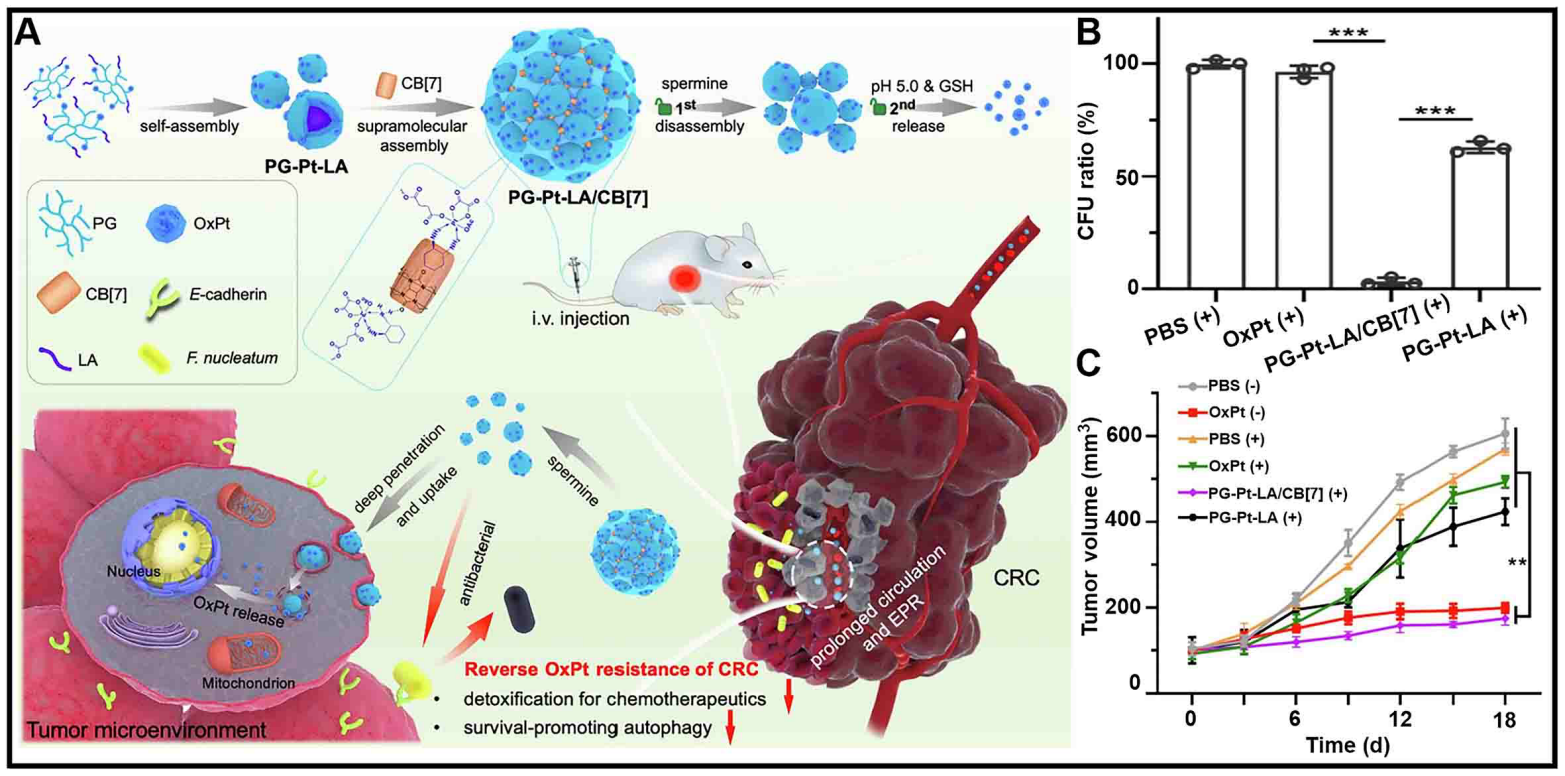
(A) Schematic illustration of the preparation and mechanism of CB[7]-based nanomedicine (PG-Pt-LA/CB[7]) in overcoming drug resistance of CRC cells; (B) Changes of *F. nucleatum* levels in CRC cells with different treatments; (C) Changes of tumor volume in tumor-bearing mice with different formulations (^**^P < 0.01, ^***^P < 0.001). This figure is quoted with permission from Yan *et al.*^[144]^. CRC: Colorectal cancer.

The *Fusobacterium nucleatum* (*F. nucleatum*) with apoptosis-inhibiting effect can trigger drug resistance in colorectal cancer (CRC) cells^[140-143]^. To address this issue, Yan *et al.* constructed a CB[7]-based nanomedicine (PG-Pt-LA/CB[7]) by multiple assemblies to overcome drug resistance [Figure 11A]^[144]^. PG-Pt-LA/CB[7] targeted and penetrated cancer cells and released OxPt in response to the GSH. The efficient uptake and stable release of drugs increased the accumulation of OxPt in CRC cells. In addition, PG-Pt-LA/CB[7] showed the best inhibition effect on *F. nucleatum* compared to OxPt and PG-Pt-LA, successfully overcoming the drug resistance of CRC cells caused by *F. nucleatum* [Figure 11B]. A negligible growth in tumor volume was observed after 18 d of incubating tumors with PG-Pt-LA/CB[7], showing that PG-Pt-LA/CB[7] improved the chemotherapeutic effect of OxPt on CRC cells [Figure 11C]. PG-Pt-LA/CB[7] was expected to be a good material for improving the effect of OxPt on CRC cells.

## PILLARARENES-BASED HOST-GUEST NANOSYSTEMS FOR OVERCOMING CANCER DRUG RESISTANCE

Pillararenes (P[*n*]As) are a new type of macrocyclic hosts that bridge hydroquinone units through methylene discovered by Ogoshi *et al.* [Figure 1D]^[145]^. These hydroquinone units are generally 5-10, with P[5]A and P[6]A being the most common^[146-151]^. P[*n*]As are widely used in various fields such as drug delivery, ion recognition, adsorptive separation, sensors, and optoelectronic materials due to the characteristics of symmetrical rigid skeleton, adjustable electron-rich cavity, and easy functionalization^[152-154]^. In addition, because of the highly attractive host-guest properties of P[*n*]As, more and more attention has been paid to the construction of P[*n*]As-based host-guest nanosystems to overcome cancer drug resistance^[155-157]^. Liu *et al.* prepared a novel carboxylatopillar[5]arene-based supramolecular quaternary ammonium nanoparticle to overcome the drug resistance generated during the chemotherapy of CRC^[158]^. Chang *et al.* constructed a redox-responsive cationic vesicle based on amphiphilic pillar[5]arene, successfully overcoming the drug resistance of tumors^[159]^.

**Figure 12:**
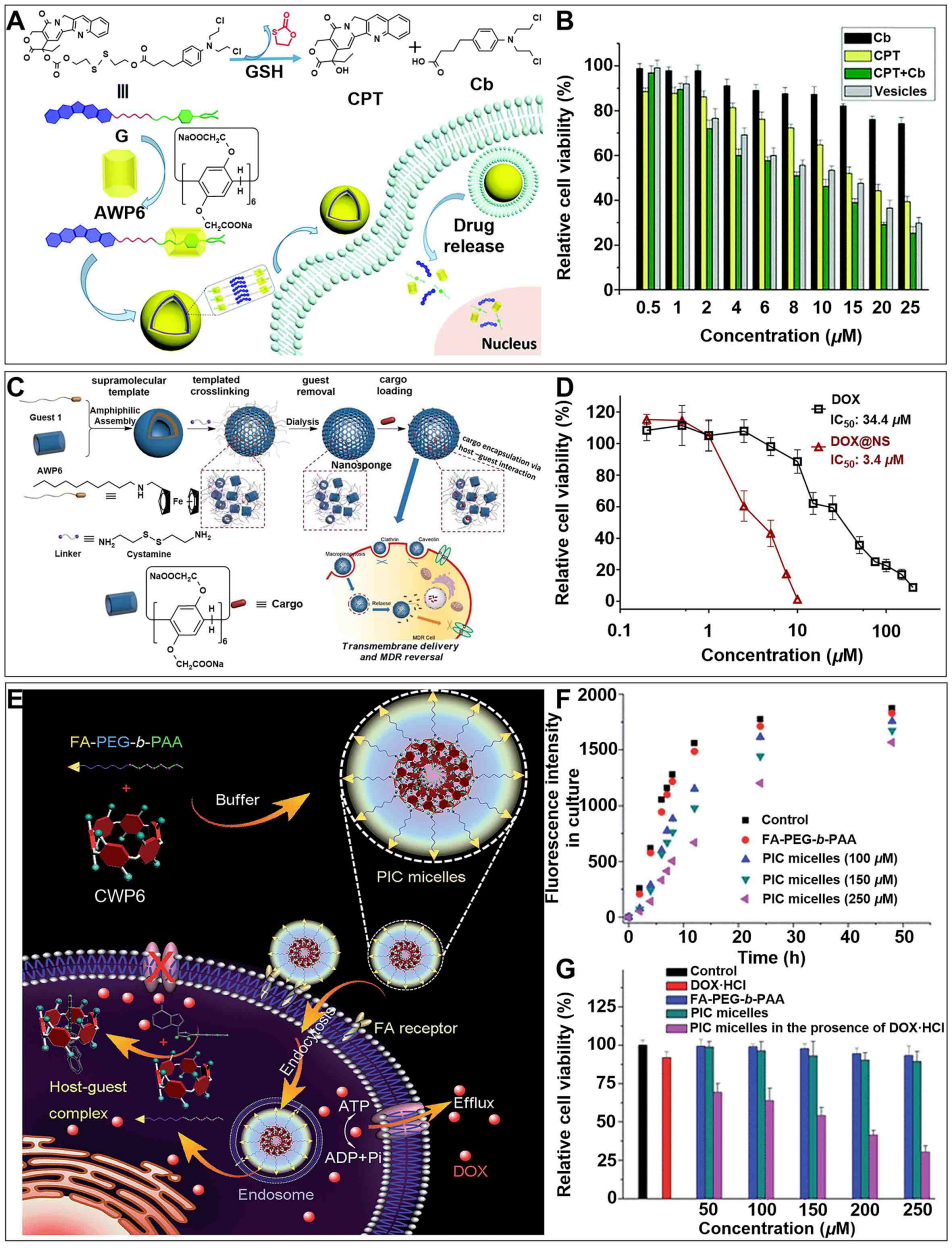
(A) Schematic illustration of the formation of nanovesicle and its internalization progress; (B) Cell viability of MCF-7 cells after incubation with Cb, CPT, Cb + CPT mixture, and vesicles for 24 h. This figure is quoted with permission from Shao *et al.*^[167]^; (C) Schematic illustration of water-solution pillar[6]arene nanosponges (NS) in overcoming MDR; (D) Cell viability of MCF-7/ADR cells after incubation with DOX and DOX@NS. This figure is quoted with permission from Liu *et al.*^[168]^; (E) Schematic illustration of the preparation of PIC micelles and their application in inhibiting drug eﬄux; (F) Changes of extracellular fluorescence intensity after incubating with FA-PEG-*b*-PAA and diﬀerent concentrations of PIC micelles; (G) Cell viability of MCF-7/ADR cells after incubation with different treatments. This figure is quoted with permission from Yu *et al.*^[170]^. AWP6: Anionic WP6; CPT: camptothecin; DOX: doxorubicin; FA: folic acid; GSH: glutathione; MDR: multidrug resistance; NS: nanosponge; PEG: poly (ethylene glycol); PIC: polyion complex.

The water-soluble pillar[6]arene (WP6) not only forms stable host-guest complexes with a variety of guest molecules but also exhibits good biocompatibility and stimuli-responsiveness, which offers the possibility for constructing supramolecular host-guest nanoplatforms to reverse cancer drug resistance^[160-166]^. Shao *et al.* reported a host-guest complex (AWP6
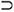
G) containing anionic WP6 (AWP6) and prodrug (G), which further self-assembled to form nanovesicles for inhibiting cancer drug resistance [Figure 12A]^[167]^. The nanovesicles released camptothecin (CPT) and chlorambucil (Cb) under the action of GSH to achieve combination chemotherapy. The dual-drug co-loaded nanovesicles showed a better inhibition effect on drug-resistance cells compared to the single drug [Figure 12B]. This study showed that P[*n*]As-based supramolecular host-guest nanosystems were expected to be ideal materials for inhibiting MDR.

Subsequently, Liu *et al.* prepared a nanosponge (NS) based on AWP6 using a “bottom-up” template preparation technique [Figure 12C]^[168]^. Through the host-guest interaction, antitumor drugs and dyes were stably encapsulated in AWP6 to overcome MDR. The IC_50_ of DOX@NS (3.4 μM) was significantly lower than that of free DOX (34.4 μM) when different doses of free DOX and DOX-loaded NS were incubated in drug-resistance cells [Figure 12D]. Mechanistic studies indicated that the effective loading and stable encapsulation of DOX based on host-guest interaction were the main reasons for overcoming MDR. This work showed that the delivery of anticancer drugs through host-guest interaction was a promising way to overcome MDR.

Additionally, cationic WP6 (CWP6) can encapsulate ATP, blocking the energy of drug efflux^[169]^. Yu *et al.* prepared a polyion complex (PIC) micelle by modifying CWP6 with functionalized diblock copolymer (FA-PEG-*b*-PAA) [Figure 12E]^[170]^. PIC micelles could specifically target and penetrate cancer cells overexpressed with FA receptors. The decrease in extracellular fluorescence intensity indicated that CWP6 successfully blocked the energy source of calcein (model drug) efflux [Figure 12F]. In addition, PIC micelles significantly enhanced the inhibitory effect of DOX·HCl on cell viability compared with free DOX·HCl [Figure 12G]. These results suggested that the supramolecular nanomicelle endocytosed by drug-resistance cells and released CWP6 to selectively form a host-guest complex with ATP, which provided a new method for blocking the energy of drug efflux and was expected to become an ideal material for overcoming cancer drug resistance.

**Figure 13:**
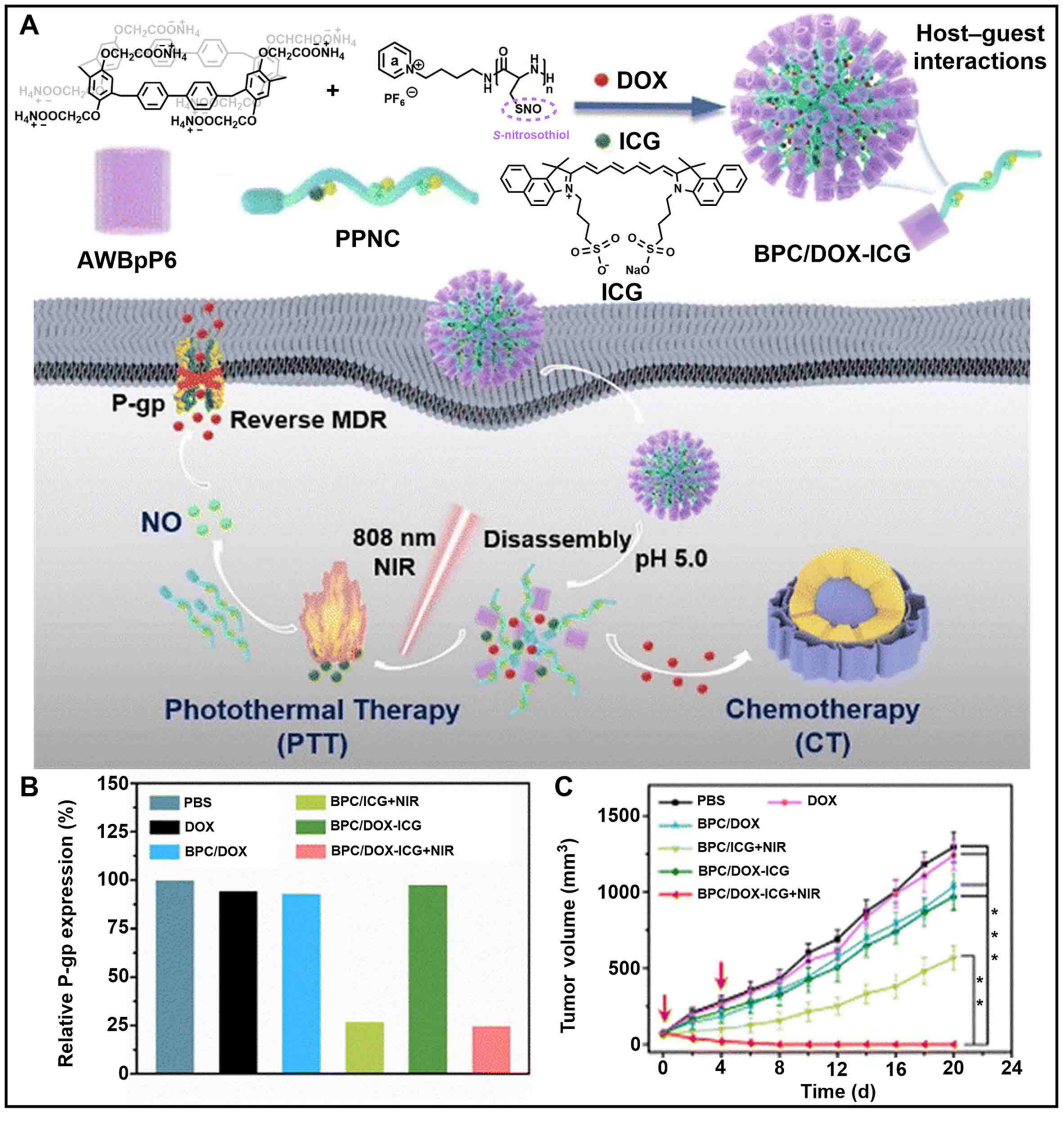
(A) Schematic illustration of synergistic PTT and CT using supramolecular polypeptide nanomedicine (BPC/DOX-ICG); (B) The P-gp expression levels in MCF-7/ADR cells with different treatments; (C) Changes of tumor volume in tumor-bearing mice with different formulations (^**^P < 0.01, ^***^P < 0.001). This figure is quoted with permission from Ding *et al.*^[174]^. DOX: Doxorubicin; ICG: indocyanine green; MDR: multidrug resistance; NIR: near-infrared; PPNC: pyridinium-terminal-modified polypeptide.

In addition to blocking the energy source of P-gp expression, nitric oxide (NO) can also downregulate the expression level of P-gp, reversing the drug resistance of cancer^[171-173]^. To achieve stable delivery and selective release of NO in tumor cells, Ding *et al.* designed supramolecular peptide nanomedicine (BPC/DOX-ICG) based on the host-guest complexation of anionic water-soluble [2]biphenyl-extended-pillar[6]arene (AWBpP6) with pyridinium-terminal-modified polypeptide (PPNC) [Figure 13A]^[174]^. DOX and indocyanine green (ICG) loaded in BPC/DOX-ICG were used to simultaneously treat cancer cells with chemotherapy and photothermal therapy. *S*-nitrosothiol on PPNC released NO to downregulate the expression level of P-gp after near-infrared (NIR) irradiation. Western Blot analysis showed that the P-gp level in MCF-7/ADR cells was significantly reduced to 24.9% when treated with NIR irradiation and BPC/DOX-ICG [Figure 13B]. The reduced P-gp could greatly enhance the efficacy of chemotherapeutic drugs, inhibiting tumor growth [Figure 13C]. Therefore, P[*n*]As-based nanocarriers could effectively deliver NO to downregulate P-gp expression, providing a promising approach to eliminate cancer drug resistance.

**Figure 14:**
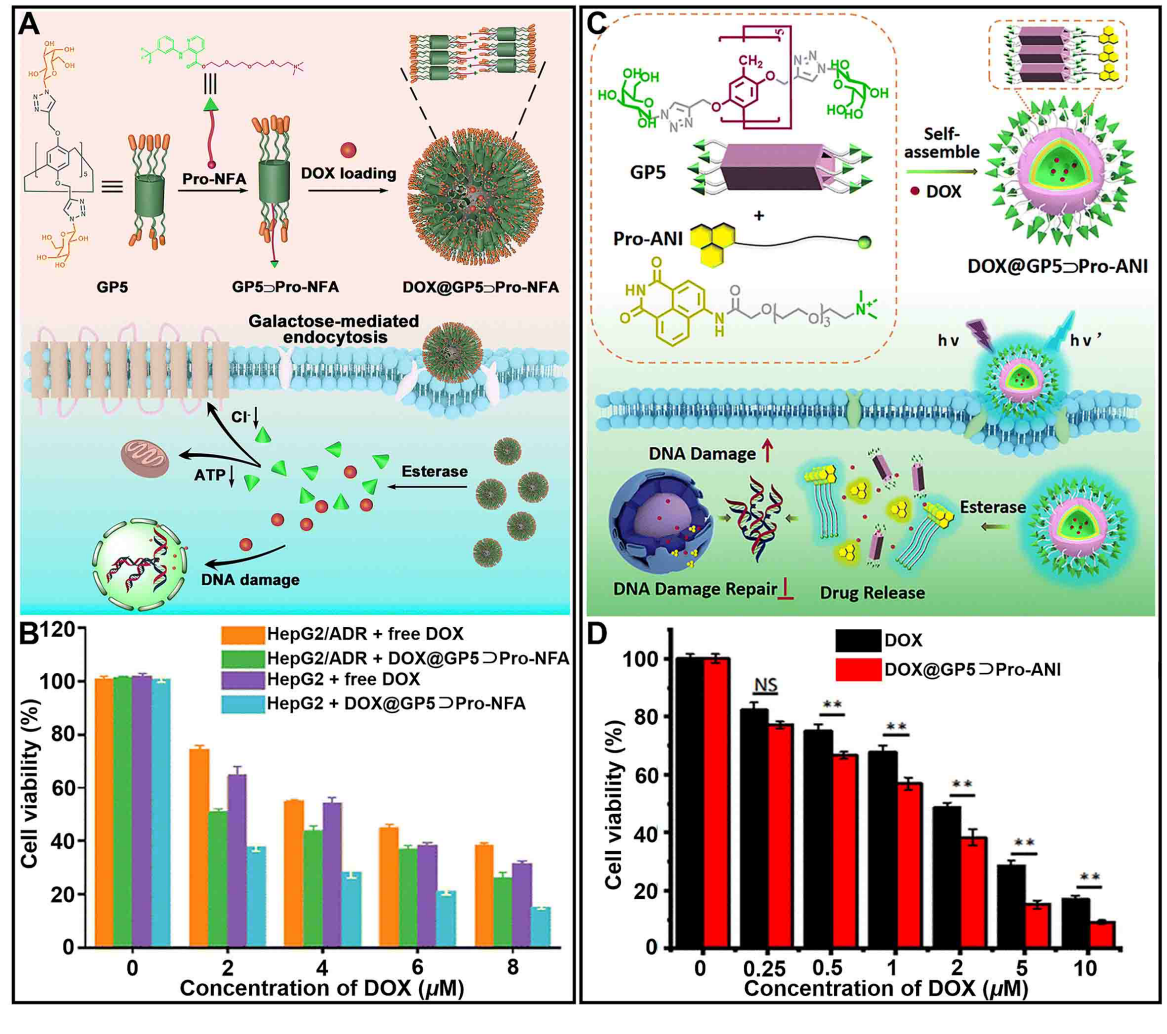
(A) Schematic illustration of the preparation of supramolecular nanoprodrugsDOX@GP5
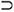
Pro-NFA and their applications in overcoming cancer drug resistance; (B) Cell viability of HepG2 cells and HepG2/ADR cells treated with free DOX and DOX@GP5
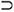
Pro-NFA, respectively. This figure is quoted with permission from Yang *et al.*^[177]^; (C) Schematic illustration of the preparation of supramolecular nanoprodrugs DOX@GP5
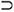
Pro-ANI and their applications in overcoming cancer drug resistance; (D) Cell viability of HepG2/ADR cells treated with free DOX and DOX@GP5
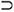
Pro-ANI (^**^P < 0.01). This figure is quoted with permission from Yang *et al.*^[180]^. DOX: Doxorubicin; GP5: galactose-modified pillar[5]arene.

Chloride channel protein is highly expressed in various cancer cells and has a significant correlation with tumor drug resistance^[175,176]^. Yang *et al.* reported a supramolecular nanoprodrug (DOX@GP5
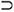
Pro-NFA) based on the host-guest complexation between galactose-modified pillar[5]arene (GP5) and chloride channel inhibitor prodrug (Pro-NFA) to reverse drug resistance [Figure 14A]^[177]^. DOX@GP5
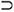
Pro-NFA was hydrolyzed under the action of esterase to release DOX and NFA, which could effectively block chloride ion channels, and reverse cancer drug resistance. Additionally, the inhibitory effect of DOX@GP5
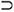
Pro-NFA on tumor cells, especially drug-resistance cells, was significantly higher than that of free DOX [Figure 14B]. Moreover, poly(ADP ribose)polymerase (PARP) can repair DNA to directly lead to drug resistance^[178,179]^. Yang *et al.* designed a nanoparticle (DOX@GP5
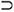
Pro-ANI) based on GP5 to load PARP inhibitor prodrug (Pro-ANI) that could inhibit DNA repair [Figure 14C]^[180]^. DOX@GP5
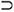
Pro-ANI overcame tumor drug resistance by inhibiting the expression of PARP, effectively reducing the viability of drug-resistance cells [Figure 14D]. These studies showed that P[*n*]As could effectively load anticancer prodrugs, which opened up broad prospects for inhibiting the expression of proteins associated with drug resistance.

## CONCLUSION

In summary, we reviewed the application of supramolecular host-guest nanosystems based on cyclodextrins, calixarenes, cucurbiturils, and pillararenes in overcoming cancer drug resistance. Compared with traditional small molecule drugs, nanosystems can effectively reduce the side effects of drugs and improve the accumulation of drugs in tumors. However, traditional nanosystems also have some drawbacks, such as lack of stimuli-responsiveness, difficulty in preparation and synthesis, and slow degradation *in vivo*. The emergence of supramolecular nanosystems complements the drawbacks of these traditional nanosystems. Due to their dynamic and reversible host-guest interactions, supramolecular nanosystems are endowed with rich stimuli-responsiveness to release drugs within tumors. Furthermore, supramolecular host-guest nanosystems have some advantages, such as high drug loading, low side effects, and good biocompatibility, which can co-deliver multiple drugs to inhibit cancer drug resistance by damaging mitochondrial function, blocking the energy source, inhibiting DNA repair, and reducing the level of GSH. However, supramolecular host-guest nanosystems still face some challenges in overcoming cancer drug resistance:

(i) Further improve the targeting. Tumor cells differ from healthy cells in many ways, such as acidity, hypoxia, and metabolism. Thus, more specific supramolecular host-guest nanosystems should be developed by focusing on the tumor microenvironment to target and penetrate tumor cells, reversing cancer drug resistance caused by drug uptake; (ii) Optimize drug loading strategy. Although multidrug-loaded supramolecular host-guest nanosystems can inhibit drug resistance, their effects are difficult to predict due to the different pharmacokinetics of each drug. Therefore, it is necessary to optimize the combination and dosage of loaded drugs and design supramolecular host-guest nanosystems with excellent performances; (iii) Improve stability. Supramolecular host-guest nanosystems have rich stimuli-responsiveness, but at the same time, there are problems of poor stability. In future studies, the stimuli-responsiveness of supramolecule and the stability of macromolecule can be better combined to prepare supramolecular polymeric nanosystems to overcome cancer drug resistance; (iv) Promote the development of cancer synergistic therapy. At present, most supramolecular host-guest nanosystems used to overcome drug resistance of tumors remain at the level of drug delivery, which greatly limits the inhibitory effect on drug-resistance cells. Introducing other therapeutic methods (such as photodynamic therapy, gene therapy, and immunotherapy) into supramolecular host-guest nanosystems will help establish more accurate and personalized strategies to combat cancer drug resistance; (v) Enhance the clinical translation. While some preliminary studies have shown that supramolecular host-guest nanosystems can be used to overcome drug resistance in cancer, there is still a long way to go before clinical translation. Firstly, the pharmacokinetics, biodistribution, metabolic behavior, and toxicological characteristics of supramolecular host-guest nanosystems are still in the research stage, and there are still unpredictable risks to their safety. Secondly, *in vitro* and *in vivo* experiments of supramolecular host-guest nanosystems cannot completely mimic the complex microenvironment of tumors in the body, resulting in lower clinical therapeutic effects than expected. Thirdly, the large-scale production of supramolecular host-guest nanosystems is a bottleneck in clinical applications, and small changes in the manufacturing process can cause significant changes in their physicochemical properties, which will affect their safety and biological effects. Therefore, more basic research and clinical trials are needed to assess their safety, efficacy, and feasibility.

The development of supramolecular host-guest nanosystems offers new hope to alleviate drug resistance in cancer, although innovation and progress are still required in many aspects. We believe that with continued research efforts, supramolecular host-guest nanosystems will make further progress in reversing cancer drug resistance, and bring new breakthroughs for cancer treatment and even human health.

## DECLARATIONS

### Copyright

© The Author(s) 2023.
